# Mitochondria-Targeted Antioxidants for Treatment of Hearing Loss: A Systematic Review

**DOI:** 10.3390/antiox8040109

**Published:** 2019-04-24

**Authors:** Chisato Fujimoto, Tatsuya Yamasoba

**Affiliations:** 1 Department of Otolaryngology and Head and Neck Surgery, Graduate School of Medicine, The University of Tokyo 7-3-1, Hongo, Bunkyo-ku, Tokyo 113-8655, Japan; tyamasoba-tky@umin.ac.jp; 2 Department of Otolaryngology, Tokyo Teishin Hospital, 2-14-23, Fujimi, Chiyoda-ku, Tokyo 102-8798, Japan

**Keywords:** antioxidants, hearing loss, mitochondria, reactive oxygen species

## Abstract

Mitochondrial dysfunction is associated with the etiologies of sensorineural hearing loss, such as age-related hearing loss, noise- and ototoxic drug-induced hearing loss, as well as hearing loss due to mitochondrial gene mutation. Mitochondria are the main sources of reactive oxygen species (ROS) and ROS-induced oxidative stress is involved in cochlear damage. Moreover, the release of ROS causes further damage to mitochondrial components. Antioxidants are thought to counteract the deleterious effects of ROS and thus, may be effective for the treatment of oxidative stress-related diseases. The administration of mitochondria-targeted antioxidants is one of the drug delivery systems targeted to mitochondria. Mitochondria-targeted antioxidants are expected to help in the prevention and/or treatment of diseases associated with mitochondrial dysfunction. Of the various mitochondria-targeted antioxidants, the protective effects of MitoQ and SkQR1 against ototoxicity have been previously evaluated in animal models and/or mouse auditory cell lines. MitoQ protects against both gentamicin- and cisplatin-induced ototoxicity. SkQR1 also provides auditory protective effects against gentamicin-induced ototoxicity. On the other hand, decreasing effect of MitoQ on gentamicin-induced cell apoptosis in auditory cell lines has been controversial. No clinical studies have been reported for otoprotection using mitochondrial-targeted antioxidants. High-quality clinical trials are required to reveal the therapeutic effect of mitochondria-targeted antioxidants in terms of otoprotection in patients.

## 1. Introduction

Many studies have been carried out under the assumption that mitochondrial dysfunction is an important pathological condition leading to hearing loss. Sensorineural hearing loss (SNHL), a type of hearing loss, occurs due to damage to the hair cells (HCs) in the cochlea and/or damage to the auditory neural pathway. Mitochondrial dysfunction is associated with the etiologies of SNHL, such as age-related hearing loss (ARHL), noise-induced hearing loss (NIHL) and ototoxic drugs, as well as hearing loss due to mitochondrial gene mutation. Mitochondria are the main sources of reactive oxygen species (ROS) [1,2]. ROS-induced oxidative stress and collapse of the redox state are involved in cochlear damage [3,4]. Moreover, the release of ROS causes further damage to mitochondrial components, such as mtDNA, mitochondrial membranes and respiratory chain proteins, as well as nuclear DNA associated with mitochondrial function [5].

Antioxidants are thought to counteract the deleterious effects of ROS and may be effective in the treatment of oxidative stress-related diseases, including part of SNHL. Recently, novel treatments based on antioxidant compounds that specifically target mitochondria have been developed for diseases associated with mitochondrial dysfunction. Mitochondria-targeted antioxidants are superior in attenuating mitochondrial oxidative damage [6,7,8]. There has also been research focusing on the application of these mitochondria-targeted antioxidants in the treatment of hearing loss [9,10,11,12,13,14,15,16].

In this systematic review, we will focus on SNHL associated with mitochondrial dysfunction and mitochondria-targeted antioxidant treatments, although a full discussion about the pathophysiology and anti-oxidant therapy in different types of SNHL is beyond the scope. First, we will briefly outline the effect of mitochondrial dysfunction on the disease. After this, we will provide an overview of hearing loss that is suggested to be associated with mitochondrial dysfunction. Finally, we will describe the application of mitochondria-targeted antioxidant treatments developed in recent years for the treatment of hearing loss.

## 2. Effect of Mitochondrial Dysfunction on Disease

Abnormalities in nuclear DNA and mitochondrial DNA (mtDNA) are observed in mitochondria-related diseases. Some of the causative genes of human diseases encode proteins associated with mitochondria. mtDNA contains 37 genes and the abnormalities of mtDNA include point mutations and structural abnormalities, such as deletions and duplications. Furthermore, novel causative nuclear DNA and mtDNA mutations have been discovered using next generation sequencing [17,18].

Mitochondria have various biological roles, such as the production of ATP, generation of ROS, involvement in apoptosis, regulation of intracellular calcium ion concentrations and protection against infection. Abnormalities of mtDNA have various effects on mitochondrial gene expression and lead to the dysregulation of oxidative phosphorylation, generation of ROS and activation of mitochondria-mediated apoptotic pathways. ROS, such as hydrogen peroxide, hydroxyl radicals, singlet oxygen and superoxide anions, are metabolized or scavenged by endogenous antioxidants, such as catalase, superoxide dismutase (Sod) and glutathione, in order to maintain cellular homeostasis [19,20]. However, aging, drug exposure and other factors can alter homeostasis. ROS generation and ROS-induced apoptosis have a large contribution to several diseases, including being a part of SNHL pathologies [3,4,20]. As far as the relationship between mitochondrial dysfunction and ROS is concerned, the mitochondrion is a primary generator of ROS, which are created as byproducts of metabolism [1,2]. ROS causes further damage to mitochondrial components, such as mtDNA, mitochondrial membranes and respiratory chain proteins, as well as nuclear DNA associated with mitochondrial function [5].

Mitochondrial abnormalities cause cell dysfunction and death. In pathological conditions caused by heteroplasmy in mtDNA, it is known that the severity of disease correlates with the level of mtDNA heteroplasmy. The mutation rate varies from cell to cell and cellular dysfunction occurs when the mutation rate exceeds the biochemical threshold [21,22]. Clinical symptoms may appear in tissues and organs where high levels of mutations have accumulated. This property is the basis for the diversity of clinical symptoms seen in mitochondria-related diseases.

## 3. Overview of Hearing Loss Associated with Mitochondrial Dysfunction

### 3.1. Hearing Loss Due to Mitochondrial Gene Mutation

Mutations in mtDNA are associated with both syndromic and non-syndromic hearing loss. A1555G in mitochondrially encoded 12S rRNA (MT-RNR1) and A3243G in mitochondrially encoded tRNA leucine 1 (UUA/G) (MTTL1) are relatively frequent mtDNA mutations associated with SNHL [23]. With regard to syndromic hearing loss, systemic neuromuscular disorders, such as mitochondrial encephalomyopathy, lactic acidosis, stroke-like episodes, mitochondrial encephalomyopathy with ragged red fibers and chronic progressive external ophthalmoplegia, frequently have sensorineural hearing loss as one of their findings [24,25,26]. Mitochondrial mutations are also found in maternally inherited diabetes and deafness [23]. Non-syndromic SNHL associated with mtDNA mutations is generally progressive and symmetric, predominantly involving the high frequency range [27]. For non-syndromic hearing loss, most of the pathogenic variants have been identified in MT-RNR1 or mitochondrially encoded tRNA serine 1 (MT-TS1) gene [27]. A1555G in MT-RNR1 is the most common non-syndromic mutation.

### 3.2. ARHL

ARHL is a progressive SNHL associated with aging. ARHL is considered to have a multifactorial etiology, including both environmental and hereditary factors. Pathological analyses show that ARHL in humans is generally classified into a loss of sensory HCs, loss of spiral ganglion neurons (SGNs) and atrophy of the stria vascularis [28,29]. Furthermore, most cases of ARHL exhibit mixed pathological changes [28].

It has been suggested that mitochondria are involved in ARHL pathology. The fast-aging senescence accelerated mouse-prone 8 (SAMP8) strain is derived from mice that have been continuously brother–sister mated to select a phenotype of accelerated senescence. This strain was used for gerontological research [30] and showed that premature ARHL is associated with sensory, strial and neural degeneration [31]. The premature ARHL in the SAMP8 strain is thought to be a result of oxidative stress, a change in the level of antioxidant enzymes and a decrease in the activity of complexes I, II and IV, leading to the activation of apoptotic cell death pathways [31]. In the SGNs of the SAMP8 strain, mitochondrial biogenesis decreased in older age when it was examined as the ratio of mtDNA/nuclear DNA and the activity of citrate synthase [31]. A short exposure to H_2_O_2_ was used to create a premature senescence model in House Ear Institute-Organ of Corti 1 (HEI-OC1) mouse auditory cells, which exhibited damage to the mitochondrial morphology, a decrease in mitochondrial membrane potential, an imbalance of mitochondrial fusion/fission and impaired mitochondrial respiratory capacity [32].

Abnormalities in mtDNA have been proposed as important factors in aging. Human temporal bone studies have reported that deletions and mutations in mtDNA in the cochlea and SGNs are increased in ARHL patients compared to that of normal hearing subjects [33,34,35]. Animal studies have also reported that an accumulation of mtDNA mutations led to premature aging. POLG D257A mice, which express an exonuclease-deficient form of the mtDNA polymerase γ, were found to accumulate mtDNA mutations more rapidly. POLG D257A mice showed a variety of phenotypes related to accelerated aging at 9–10 months old, including the early onset of ARHL. In the basal turn of the cochlea, 9–10 month old POLG D257A mice showed a severe loss of SGNs and HCs and significant elevation of apoptosis [36].

Peroxisome proliferator-activated receptor gamma coactivator 1-α (PGC-1α) is a transcription coactivator that regulates mitochondrial biogenesis and energy metabolism, which is also suggested to be associated with aging. A decrease in PGC-1α is a key pathological factor of age-related diseases [37]. The overexpression of PGC-1α in a rat model with strial marginal cell senescence harboring the mtDNA4834 common deletion caused a significant decrease in mtDNA deletion and apoptotic cells in the cochlea [38].

The increase in ROS associated with the progression of aging is closely related to the development of ARHL. Sod is an antioxidant enzyme that catalyzes the dismutation of the superoxide anion into oxygen and hydrogen peroxide. *Sod1* knockout mice showed premature ARHL with cochlear HC loss, reduction in strial thickness and degeneration of SGNs [39,40]. Glutathione peroxidase (GPX) is an antioxidant enzyme that reduces hydrogen peroxide to water and lipid peroxides to lipid alcohols and oxidizes glutathione to glutathione disulfide. Increased GPX activity was observed in the stria vascularis and spiral ligament of the cochlea in aged rats [41]. *Gpx1* knockout mice exhibited greater hearing thresholds at high frequencies and severe cochlear HC loss [42]. BCL2-antagonist/killer 1 (*Bak*), a mitochondrial proapoptotic gene, is induced by oxidative stress and *Bak* deficiency prevents apoptotic cell death. *Bak* knockout mice exhibited reduced apoptotic cell death in both SGNs and cochlear HCs, leading to the prevention of ARHL [43]. Isocitrate dehydrogenases (IDHs) are enzymes that catalyze the oxidative decarboxylation of isocitrate to α-oxoglutarate. IDH2 is a mitochondrial NADP^+^-dependent enzyme that plays a key role in mitochondrial antioxidant systems through the generation of NADPH. *Idh2* knockout male mice showed accelerated ARHL and this phenotype was accompanied by an increase in oxidative DNA damage, increased apoptotic cell death and profound loss of SGNs and cochlear HCs [44].

### 3.3. NIHL

NIHL is a type of SNHL caused by exposure to loud noise. Generally, the outer HCs (OHCs) are more sensitive to noise compared to the inner HCs (IHCs). Damage following noise exposure can be categorized into three main types: mechanical damage, neural degeneration and metabolic damage [45]. Mitochondria are suggested to be involved in the metabolic damage caused by acoustic trauma. Morphologically, mitochondrial damage was observed in the OHCs of guinea pigs after noise exposure [46] and the damage was localized in mitochondrial cristae in HCs 2 h after noise exposure.

Dysregulation of Ca^2+^ buffering in mitochondria and/or the cytosol can be part of the pathophysiology of NIHL. An elevation of the calcium level in the cytosol was observed in HCs after noise exposure [47,48]. Noise exposure caused the activation of calcineurin, which is activated by the binding of Ca^2+^/calmodulin, in cochlear HCs [49] and led to mitochondria-mediated apoptosis [50]. The mitochondrial calcium uniporter (MCU) is a major specific calcium channel for calcium uptake from the cytosol into mitochondria [51] and the overexpression of MCU renders cells susceptible to apoptotic cell death [52]. Noise exposure caused an overexpression of MCU in HCs of the cochlear basal turn [53]. Inhibiting MCU resulted in the attenuation of NIHL with prevention of noise-induced OHC loss and noise-induced loss of IHC synaptic ribbons [53].

Noise exposure has been reported to increase ROS generation in cochlear fluids and tissues, including HCs and the stria vascularis [54,55,56]. It is assumed that the exposure to noise induces mitochondrial damage and thereby increases ROS production. In addition, an elevation of calcium stimulates respiratory chain activity, leading to an increase in ROS [57]. Antioxidant enzymes play an important role in protecting the cochlea against noise trauma. *Gpx1* knockout mice exhibited greater HC loss as a result of noise exposure and exacerbation of NIHL [42]. *Sod2* heterozygous knockout mice also exhibited greater damage of OHCs by noise exposure and exacerbation of NIHL [58].

Noise exposure has been shown to induce the production of lipid peroxidation products, such as 8-iso-prostaglandin F2α in the cochlea [59]. Furthermore, 8-iso-prostaglandin F2α is a potent vasoconstrictor and is thought to contribute to a decrease in cochlear blood flow due to noise exposure [60]. Excessive noise exposure reduced capillary blood flow and caused vasoconstriction [60,61]. Ischemia may reduce the oxygen supply to the tissue and decrease ATP levels in the cochlea, including in the lateral wall that contributes to the maintenance of the fluid and ion homeostasis [62,63,64]. Therefore, a decrease in ATP levels is involved in NIHL. In noise exposure, damage to the mitochondria, the main ATP source, may also contribute to the decreased ATP levels.

### 3.4. Ototoxic Hearing Loss

There are two types of well-known ototoxic drugs in clinical settings, namely aminoglycoside and platinum-based anticancer drugs. Aminoglycoside ototoxicity causes preferential high-frequency hearing loss and preferential OHC loss from the basal turn of the cochlea. Ototoxicity induced by cisplatin, a representative platinum-based anticancer drug, can also lead to preferential high-frequency hearing loss and preferential OHC loss from the basal turn.

Mitochondrial dysfunction has been suggested to play an important role in aminoglycoside-induced HC injury. Inhibition of mitochondrial protein synthesis with chloramphenicol significantly increased gentamicin-induced auditory HC death [65,66]. Gentamicin application induced a loss of mitochondrial membrane potential in OHCs before the occurrence of HC death [65]. A decrease in NADH formation was observed shortly after gentamicin application and this effect was most pronounced in OHCs at the basal turn of the cochlea, suggesting that gentamicin rapidly inhibits mitochondrial metabolism [67]. Inhibiting tRNA 5-methylaminomethyl-2-thiouridylate methyltransferase (TRMU), a mitochondrial tRNA-modifying enzyme, increased the sensitivity of HEI-OC1 auditory cells to neomycin damage [68]. The mitochondrial ribosome has been proposed as a target in aminoglycoside ototoxicity. Genetically, A1555G and C1494T mutations of the MT-RNR1 gene have been shown to be linked to aminoglycoside hypersensitivity [69]. Gentamicin also directly inhibits protein synthesis in mitochondrial ribosomes [70].

Aminoglycoside ototoxicity is also associated with the induction of oxidative stress. Gentamicin-induced ROS generation in cochlear explants has been detected using electron paramagnetic resonance spectroscopy [71]. Toxic levels of ROS led to apoptotic HC death [72,73]. When aminoglycoside was combined with iron salts, the aminoglycoside–iron complex directly promoted the formation of ROS [74,75,76]. In addition, ROS generation in the inner ear has been suggested to be associated with enzymatic reactions catalyzed by nitric oxide synthase [77] and NADPH oxidase [78]. Furthermore, the above-mentioned mitochondrial dysfunction due to aminoglycoside might elevate ROS levels.

ROS is also suggested to play an important role in cisplatin-induced ototoxicity. Cisplatin application increased the generation of ROS [79]. NADPH oxidase 3 and xanthine oxidase has been proposed to be ROS generating systems for cisplatin in the cochlea [80,81,82]. The pathophysiology of cisplatin-induced oxidative stress in the cochlea includes glutathione depletion [83,84,85], reduced antioxidant enzyme activity [84,85] and increased lipid peroxidation [86].

Mitochondrial apoptotic pathways are also involved in the ototoxicity after cisplatin application. Cisplatin application led to an increased expression of BCL2-associated X protein (BAX), a pro-apoptotic protein and decreased expression of BCL2, anti-apoptotic protein, in the cells of the organ of Corti, SGNs and the cochlear lateral wall in gerbils [87]. Cochlear HCs in cisplatin-treated guinea pigs showed activation and redistribution of BAX and release of cytochrome c from the mitochondria in the course of apoptotic cell death [88]. Cisplatin-treated auditory cell lines showed the activation of pro-apoptotic Bcl-2 family members and inactivation of anti-apoptotic Bcl-2 family members [89,90].

## 4. Mitochondria-Targeted Antioxidants for Treatment of Hearing Loss

### 4.1. Treatment Effect of Antioxidants on Hearing Loss

The effect of antioxidants on various diseases related to oxidative stress, including some forms of SNHL, has been studied in animal experiments and human studies. Most studies have reported that certain antioxidants can prevent ARHL but the role of antioxidants in the prevention of ARHL is still controversial [91]. For example, vitamin intake has been found to positively contribute to the prevention of ARHL in human cross-sectional studies [92,93,94,95,96], while there were no significant effects in some prospective human studies [97,98]. It is difficult to equalize the design of every human study, such as the selection of the target population and determination of hearing loss. Several interventional studies have been conducted on animals but the influence of the administration period and dose on the results should also be taken into consideration in animal experiments. In addition, some antioxidants displayed different effects between human and animal studies. In animal experiments using rats, antioxidant treatment with vitamins E, C, melatonin, lazaroids or lecithin resulted in better auditory sensitivity and less mtDNA deletions [99,100]. Clinical studies in humans showed that the hearing loss at low frequencies was slightly attenuated by the administration of vitamin C and rebamipide [101]. However, vitamin C supplementation did not elevate vitamin C levels in the cochlea or slow ARHL in mice [102]. In a study investigating the effects of 17 antioxidant compounds (acetyl-L-carnitine (ALCAR), α-lipoic acid, β-carotene, carnosine, coenzyme Q10 (CoQ10), curcumin, d-α-tocopherol, epigallocatechin-3-gallate (EGCG), gallic acid, lutein, lycopene, melatonin, N-acetyl-L-cysteine (NAC), proanthocyanidin, quercetin, resveratrol and tannic acid) on the prevention of ARHL in mice, ARHL was almost completely prevented by treatment with α-lipoic acid and CoQ10; partially prevented by NAC treatment; and not prevented by the other compounds [43]. Other studies also support the positive preventive effects of α-lipoic acid and CoQ10 on ARHL through research in laboratory animals or human elderly subjects [101,103,104,105]. On the contrary, there are differences in opinion as to whether NAC is effective for the prevention of ARHL in mice [106,107]. Folic acid intake for three years slightly slowed ARHL in a long-term double-blind, placebo-controlled human trial [108].

A number of studies have evaluated the attenuating effects of antioxidants on NIHL. Animal models have been used to assess whether reducing the effects of oxidative stress with antioxidants has any therapeutic value. For many antioxidants, such as glutathione [109,110], D-methionine [111], ebselen [112,113,114], resveratrol [115], vitamin C [116,117], water soluble CoQ10 formulation (Q10 terclatrate, Q-ter) [118,119], idebenone [120], ALCAR [121,122], ferulic acid [123], NAC [121,124,125,126,127,128,129] and hydroxylated α-phenyl tert-butylnitrone [130], the attenuation effect of NIHL and/or noise-induced cell injury in the cochlea has been reported using animal models. Out of these antioxidants, namely NAC, is most widely studied for its ability to attenuate NIHL under a variety of conditions in animal models. Many studies reported that NAC was an effective antioxidant to prevent NIHL [121,124,125,126,127,128,129] but a few studies did not show that NAC was able to provide otoprotection [131,132]. In human studies, oral administration of NAC during noise exposure prevented the noise-induced temporary threshold shift (TTS) [133], while another study showed that oral administration of NAC before loud noise exposure did not provide otoprotection [134]. A large military group exposed to loud noise showed a slight reduction in TTS after daily oral NAC administration [135]. Recently, the safety and efficacy of ebselen for the prevention of noise-induced TTS in young adults was reported in a phase 2 clinical trial [136].

With regard to ototoxicity due to aminoglycosides, many studies have been conducted on the anti-ototoxic effects of antioxidants. In animal studies, several antioxidants, such as NAC [137,138,139], D-methionine [140,141], edaravone [142], L-carnitine [143], pentoxifylline [144,145] and Q-ter [146], have been shown to reduce aminoglycoside-induced ototoxicity. Recently, a study using cochlea explant culture examined the preventive effect of a library of 81 antioxidants on HC damage induced by gentamycin [147]. Thirteen antioxidants showed significant HC protection and six showed significant harmful effects on HCs. Out of the 13 antioxidants’ protective effects, 8 have previously been shown to protect HCs against oxidative stress, including idebenone, resveratrol, DL-α-lipoic acid, MC-186, pro Cysteine, trolox, thiourea and thymoquinone. In contrast, seratrodast, butylated hydroxyanisole, hinokitiol, butylated hydroxytoluene and dithiothreitol did not reduce HC damage. The remaining antioxidants, including antioxidants that have previously been shown to protect hearing and HCs from aminoglycosides, did not show protective effects in HC. Results may vary depending on experimental conditions, such as the dose of gentamicin. In human studies, aspirin was shown to protect hearing in adults treated with gentamicin [148] and several studies demonstrated that NAC protected the hearing of patients undergoing dialysis when co-administered with either gentamicin or amikacin [149,150,151,152].

Various types of antioxidants have been reported to attenuate cisplatin-induced ototoxicity in experiments using animal models or cell lines. In investigating the protective effect of antioxidants against cisplatin-induced ototoxicity, it is also necessary to discuss whether the antioxidant has any effect on the antitumor properties of cisplatin. Studies using thiol compounds that have high affinities for platinum have long been conducted. Previous studies showed that NAC and sodium thiosulfate (STS) provided protection against cisplatin-induced ototoxicity in animal models [153,154,155] but the systemic administration of these drugs with cisplatin led to a loss of the antitumor activity of cisplatin [156]. Therefore, local administration of STS into the cochlea was necessary to obtain the otoprotection effect without attenuating the antitumor activity of cisplatin [157]. D-methionine protected against cisplatin-induced hearing loss in both systemic and topical administration [158,159]. Other antioxidants that showed protective effects against cisplatin-induced HC damage and/or hearing loss include ebselen, lipoic acid, diethyldithiocarbamate, 4-methylthiobenzoic acid, A1 adenosine receptor agonists and EGCG [89,160,161]. High doses of amifostine also showed an otoprotective effect in hamsters but it was associated with neurotoxicity [162]. In human studies, a randomized controlled trial showed that tympanic injections of STS gel were ineffective against cisplatin-induced ototoxicity [163], which was different from the results derived from animal experiments. The effect of amifostine on cisplatin-induced ototoxicity has been controversial in human studies and it is believed that this effect depends on dose [164,165,166]. It is also controversial as to whether intratympanic NAC injection is effective in protecting against cisplatin-induced ototoxicity [167,168]. A preliminary randomized, placebo-controlled study revealed that vitamin E protected against cisplatin-induced ototoxicity [169].

The majority of the above-mentioned reports are the result of research in animal models and the evidence in humans is poor. Further clinical studies are necessary to confirm the protective effects of antioxidants in various types of SNHL.

### 4.2. Mitochondria-Targeted Antioxidants for Treatment of Hearing Loss

Conventional antioxidants do not accumulate preferentially in mitochondria and therapies using these antioxidants may not achieve an effective concentration at the sites of oxidative stress due to lack of specificity for diseased organelles. Ideally, antioxidants are desired to remove only the amount produced in excess without suppressing ROS that are important for signal transduction. Recently, mitochondrial targeting of bioactive molecules has been developed and studies of mitochondria-targeted approaches for the treatments of mitochondria-related diseases, such as neurodegenerative diseases, have been conducted [170,171,172]. Mitochondria-targeted antioxidants are expected to be useful for the prevention and/or treatment of diseases associated with mitochondrial dysfunction. Mitochondria-targeted antioxidants are mainly categorized as lipophilic cation-based antioxidants, such as MitoQ [8], MitoVitE [173], MitoPBN [174], MitoPeroxidase [175], SkQ1 [176] and SkQR1 [177] or amino acid- and peptide-based antioxidants, such as SS tetrapeptides [178]. Recently, CoQ10 packed in the lipid phase of a MITO-Porter, a liposome-based mitochondrial delivery system (CoQ10-MITO-Porter), was developed as a novel mitochondria-targeted antioxidant [179]. The ability of these antioxidants to target the mitochondria is attributed to their ability to cross the phospholipid bilayer and eliminate ROS inside the mitochondria. Mitochondria-targeted antioxidants are far superior to non-targeted cellular antioxidants in reducing mitochondrial oxidative damage [6,7,8].

A systematic review was conducted in order to identify eligible articles reporting mitochondria-targeted antioxidants for treatment of hearing loss. We searched PubMed MEDLINE database published until April 22, 2019. A query-based PubMed search was performed to identify the most relevant studies reported in English. The following query was used: (((((auditory) OR inner ear) OR hearing) OR hearing loss)) AND ((((antioxidant) OR antioxidants)) AND ((mitochondria targeted) OR mitochondria target)). We screened all 20 identified records and removed all studies that were not relevant to the subject matter. The remaining 8 records were summarized in this section [9,10,11,12,13,14,15,16]. Among the mitochondria-targeted antioxidants, MitoQ and SkQR1 have been used for studying the treatment of SNHL (Table 1).

MitoQ is a derivative of CoQ10 linked to a triphenylphosphonium (TPP) cation [8]. This compound consists of the quinone moiety of CoQ and a shortened side chain linked to TPP. CoQ10 is crucial for mitochondrial respiratory chain activity and acts as an electron carrier and an antioxidant that prevents lipid peroxidation. CoQ10 alone is hydrophobic and its accumulation within mitochondria is limited [180,181]. On the contrary, the hydrophilicity and positive charge of the TPP cation in MitoQ enables its accumulation to increase by several hundred-fold within negatively charged mitochondria [173,182,183]. MitoQ could not restore respiration in ubiquinone-deficient mitochondria because the oxidation of MitoQ by complex III was poor, suggesting that MitoQ was an ineffective substrate for complex III [184]. MitoQ was also an ineffective substrate for complex I but was extensively reduced by complex II [184]. Therefore, MitoQ can act as a more effective antioxidant due to its rapid reduction and poor oxidation. The protective effect of MitoQ against oxidative stress has been reported in animal models and humans by many researchers and clinical trials related to the antioxidant effect of MitoQ have also been conducted [185]. A randomized, placebo-controlled, double-blind trial showed that MitoQ did not slow the progression of Parkinson’s disease [186]. Another randomized, placebo-controlled phase II trial revealed that MitoQ decreased liver damage in hepatitis C patients [187]. Additionally, another randomized, placebo-controlled, double-blind crossover trial revealed that MitoQ improved vascular function in healthy elderly adults [188].

SkQ1 is produced by replacing the methoxy groups on the quinone moiety of MitoQ with methyl groups [176]. SkQR1 is produced by replacing the TPP cation of SkQ1 with rhodamine 19. SkQ1 and SkQR1 have greater permeability to cross the phospholipid bilayer membrane than MitoQ [177] and have high antioxidant effects in cells and in animal models [189,190].

There have been several reports using MitoQ and SkQR1 to demonstrate the protective effect of mitochondria-targeted antioxidants against ototoxicity in animal models and/or mouse auditory cell lines [9,10,11,12,13,14,15,16]. Oral MitoQ treatment reduced gentamicin-induced cochlear damage and hearing loss in guinea pigs [15]. Treatment with SkQR1 also protected against gentamicin-induced ototoxicity in rats [11]. A study using a HEI-OC1 mouse auditory cell line demonstrated that preincubation with MitoQ significantly decreased gentamicin-induced apoptosis compared to idebenone, a short chain CoQ analogue [14]. In contrast, another study showed that MitoQ did not decrease gentamicin-induced cell apoptosis in the HEI-OC1 cell line. It attenuated gentamicin-induced upregulation of the *Bak* gene but not the Bak protein [10]. In HEI-OC1 cells, co-incubation with low-dose gentamicin and MitoQ decreased mitochondrial membrane potential, whereas co-incubation with high-dose gentamicin and MitoQ hyperpolarized the mitochondrial membrane, suggesting that the combination of gentamicin and MitoQ has the potential to disrupt the mitochondrial membrane [13]. Oral or transcutaneous MitoQ administration provided limited protection against amikacin-induced hearing loss and cochlear damage in guinea pigs [9]. Subcutaneous MitoQ administration significantly reduced cisplatin-induced OHC loss and hearing loss in guinea pigs [16]. In a recent study investigating the protective potential of MitoQ against ROS-induced cochlear damage in *Idh2* knockout mice [12], IDH2 deficiency increased the sensitivity of HCs to H_2_O_2_-induced oxidative stress and HC degeneration in *Idh2*-deficient cochlear explants. This was completely prevented by MitoQ pre-treatment, suggesting that the mitochondria-targeted antioxidative effect of MitoQ protects HCs against H_2_O_2_-induced ototoxicity even in cells deficient in IDH2. No clinical studies have been able to demonstrate the otoprotective effects of mitochondria-targeted antioxidants. Clinical trials are required to confirm the therapeutic effects of mitochondria-targeted antioxidants on otoprotection in patients.

## Figures and Tables

**Table 1:** Summary of major findings in the effect of mitochondria-targeted antioxidants on SNHL.

Antioxidants	Major Findings
Mito Q 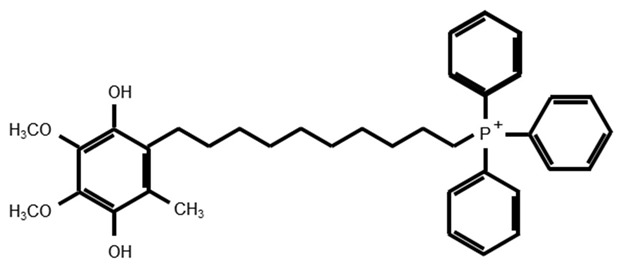	Reducing cochlear damage and hearing loss (gentamicin, guinea pig) [15]. Decreasing apoptosis as compared to idebenone (gentamicin, HEI-OC1) [14]. Not decreasing apoptosis (gentamicin, HEI-OC1) [10]. Having a potential to disrupt the mitochondrial membrane (gentamicin, HEI-OC1) [13]. Limited protection against hearing loss and cochlear damage (amikacin, guinea pig) [9] Reducing OHC loss and hearing loss (cisplatin, guinea pig) [16] Protecting HCs against ototoxicity (H_2_O_2_, IDH2-deficient cochlear explant) [12]
SkQR1 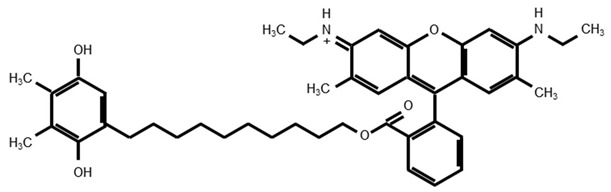	Protecting against ototoxicity (gentamicin, rat) [11].

SNHL, sensorineural hearing loss, HEI-OC1, House Ear Institute-Organ of Corti 1 cell line, IDH2, Isocitrate dehydrogenase 2.
